# Wearable Health Devices—Vital Sign Monitoring, Systems and Technologies

**DOI:** 10.3390/s18082414

**Published:** 2018-07-25

**Authors:** Duarte Dias, João Paulo Silva Cunha

**Affiliations:** 1 Biomedical Research and INnovation (BRAIN), Centre for Biomedical Engineering Research (C-BER), INESC Technology and Science, Porto 4200-465, Portugal; jpcunha@fe.up.pt; 2 Faculty of Engineering, University of Porto, Porto 4200-465, Portugal

**Keywords:** wearable health devices, vital signs, wearable systems, health, clinical, medicine, activity

## Abstract

Wearable Health Devices (WHDs) are increasingly helping people to better monitor their health status both at an activity/fitness level for self-health tracking and at a medical level providing more data to clinicians with a potential for earlier diagnostic and guidance of treatment. The technology revolution in the miniaturization of electronic devices is enabling to design more reliable and adaptable wearables, contributing for a world-wide change in the health monitoring approach. In this paper we review important aspects in the WHDs area, listing the state-of-the-art of wearable vital signs sensing technologies plus their system architectures and specifications. A focus on vital signs acquired by WHDs is made: first a discussion about the most important vital signs for health assessment using WHDs is presented and then for each vital sign a description is made concerning its origin and effect on heath, monitoring needs, acquisition methods and WHDs and recent scientific developments on the area (electrocardiogram, heart rate, blood pressure, respiration rate, blood oxygen saturation, blood glucose, skin perspiration, capnography, body temperature, motion evaluation, cardiac implantable devices and ambient parameters). A general WHDs system architecture is presented based on the state-of-the-art. After a global review of WHDs, we zoom in into cardiovascular WHDs, analysing commercial devices and their applicability versus quality, extending this subject to smart t-shirts for medical purposes. Furthermore we present a resumed evolution of these devices based on the prototypes developed along the years. Finally we discuss likely market trends and future challenges for the emerging WHDs area.

## 1. Introduction

Wearable Health Devices (WHDs) are an emerging technology that enables continuous ambulatory monitoring of human vital signs during daily life (during work, at home, during sport activities, etc.) or in a clinical environment, with the advantage of minimizing discomfort and interference with normal human activities [1].

WHDs are part of personal health systems, a concept introduced in the late 1990s, with the purpose of placing the individual citizen in the center of the healthcare delivery process, managing its own health and interacting with care providers—a concept that is commonly referred to as “patient empowerment. The aim was to raise people interest about their health status, improving the quality of care and making use of the new technology capabilities. These devices create a synergy between multiple science domains such as biomedical technologies, micro and nanotechnologies, materials engineering, electronic engineering and information and communication technologies [2,3].

The use of WHDs allows the ambulatory acquisition of vital signs and health status monitoring over extended periods (days/weeks) and outside clinical environments. This feature allows acquiring vital data during different daily activities, ensuring a better support in medical diagnosis and/or helping in a better and faster recovering from a medical intervention or body injury. WHDs are also very useful in sport activities/fitness to monitor athlete’s performance or even in first responders or military personnel to evaluate and monitor their body response in different hazardous situations and to better manage their effort and occupational health. These devices can be for both medical and/or activities/fitness/wellness purposes, always targeting the human body monitoring. Taking this in account, the best terminology is “health”, leading to WHDs. WHDs denomination can be more specific referring to which areas they are applied to. Independently of WHDs purpose, there are four main requirements on their design: low power consumption, reliability and security, comfort and ergonomics [4,5].

According to Statista [6], the wearable devices market is currently having a worldwide revenue of around $26 billion, and is expected to reach almost $34 billion in 2019. Regarding healthcare and medical environments, it is expected to grow almost to $15 billion worldwide value in 2019 [7].

This review aims to gather recent information on WHDs and better evaluate the current situation of such devices, foreseeing their evolution in the coming years. The main focus will be in vital signs and in textile embedded WHDs.

The document is organized in seven sections. In Section 2 we discuss what really are the most needed measurements to be acquired concerning medical, personal healthcare, fitness/wellness and sport activities areas. The signs where a higher technological development effort is being made is also covered. Then, Section 3 presents recent technologies used to acquire each identified vital sign. In Section 4, a generic system architecture is presented to better understand WHDs components, workflow and the differences between existing devices. Section 5 reviews some types of WHD with a main focus on heart activity monitoring devices. A focus on commercialized WHDs t-shirts is made to compare devices and discuss properties. Some prototypes are also briefly presented. To foresee possible future trends in this area, this review is complemented with a market analysis in Section 6. Finally, Section 7 concludes this review referring to some future challenges and perspectives in the WHDs area.

This review differs in several aspects from other publications with similar topics, correlating different areas and aspects related with WHDs that are sometimes not combined nor analysed. For example, in recent publications (e.g., [8,9]) there is a very good description of the technical aspects related with the physiologic signs description, acquisition methods, some devices and fabrication methods, but few information about the concerns and needs of those signs on the human health or about the WHDs system architecture. On the other hand, some other publications are strongly focused on the WHDs architecture and technical aspects of it, such as in [10]. There are still other publications that focus on other aspects, but here we present a concatenation of the main aspects related with WHDs. This review also addresses two topics that are not very much explored in WHDs technology publications which are the understanding of the most important vital signs, where some medical aspects are included, and also a market trend analysis to understand what can be the future of these type of devices. A unique characteristic of this review is the heart activity monitoring focus that is performed to understand the state-the-art of one of the largest area of WHDs.

## 2. Vital Signs—Most Important to be Monitored

**Figure 1:**
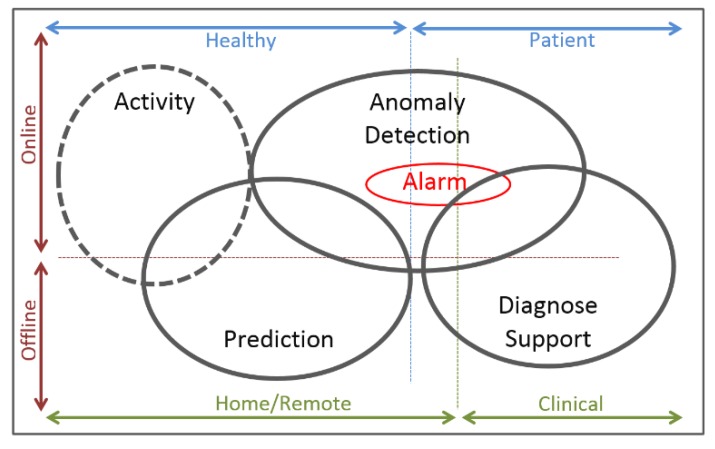
Schematic overview of the four main data mining processes (activity, prediction, anomaly detection and diagnose/decision support) in relation to different aspects of wearable sensing in wearable health devices. Filled line- Medical purposes; Traced line: Activity purposes. Adapted from [11].

The human body has multiple different physiological signs that can be measured: from electrical signs to biochemical, human biosignals are possible to be extracted and be used to better understand the bodily health status and reaction to external factors. Before understanding how the signs are produced and how they can be acquired using wearable sensors and devices, it is of major importance to understand the main biosignals that contributes for a better human body health analysis. Nowadays technology and wearable scenarios let us to classify WHDs according to three aspects (Figure 1): scenario of use (home/remote or clinical environment); the type of monitoring (offline or online); and the type of user (healthy or patient) [11].

Regarding this classification, it is possible to divide WHDs in two main areas, activity monitoring area (1) and the medical area (2) that is divided in three main sub-categories (Figure 1): 

(1) Activity area—where fitness/wellness and non-medical applications, self-monitoring and rehabilitation procedures are included.

(2.1) Prediction—consists in the identification of events that have not occurred yet, providing medical information to help in the prevention of further chronic problems, and sometimes, can support a diagnosis decision [11];

(2.2) Anomaly Detection—responsible for the identification of unusual patterns that are not conformed to the expected behaviour, based on classification methods to distinguish normal data from outlier data. Alarm is a subtask mainly used in anomalies detection, raising an alarm as soon as an anomaly is detected [11];

(2.3) Diagnosis Support—is one of the most important tasks of clinical monitoring, resulting in a clinical decision according to retrieved knowledge of vital signs, health records and anomaly detection data [11]. 

This categorization of WHDs let us better understand the main areas of application, showing their multi-purpose usages. It is known that the use and monitoring capabilities are limited to which signals WHDs are capable do capture. This will be an important topic discussed along this review.

From all the possible variables to extract from the human body we need to identify the most useful, both medical and not medical (described as “activity”), and on which the largest effort in development was done. To achieve this, and to better understand the evolution of scientific investigation in WHDs, we have performed a survey in the *Web of Science*^TM^ main collection with the criteria presented in Table 1. The search was conducted with a main topic—Wearable Device, and for two different periods, between 2010–2013 and 2014–2017. For each analysed period, we searched for “pairs” of purpose/vital sign for all combinations, excluding all others. The following description shows an example of a search: “Topic: (Wearable Device) AND Topic: (Medical) NOT Topic: (Activity) AND Topic: (Body Temperature) NOT Topic: (Blood Pressure) NOT Topic: (Respiration) NOT Topic: (Glucose) NOT Topic: (Heart Rate) NOT Topic: (oxygen saturation) NOT Topic: (Electrocardiogram); Timespan: 2010–2013”.

**Figure 2:**
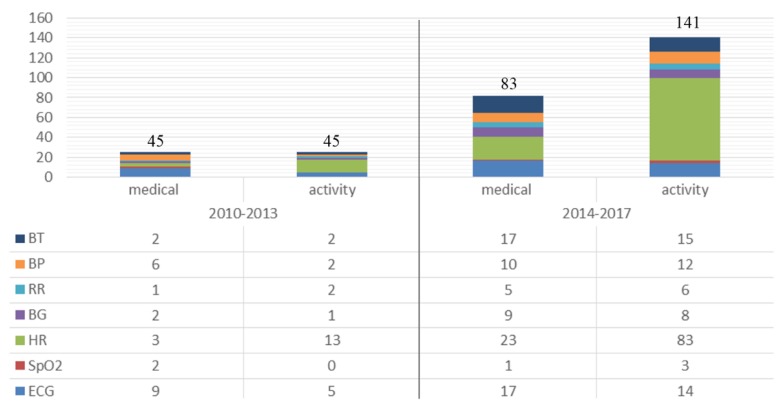
Number of retrieved scientific papers related with WHDs and a specific physiological sign. The distribution is divided in two time intervals and according to the purpose: medical or activity. (BT—Body Temperature; BP—Blood Pressure; RR—Respiration Rate; BG—Blood Glucose; HR—Heart Rate; SpO2—Blood Oxygen Saturation; ECG—Electrocardiogram).

The vital signs presented in this search where chosen according to the most frequent vital signs in the retrieved literature and they are divided into the two main areas (medical and activity) to better understand where the higher effort is being made in the research community (Figure 2). 

The medical literature points to different sets of valuable vital signs to be monitored in order to identify clinical deterioration. Overall, we have identified five traditional vital signs that have a major importance to be measured: heart rate, blood pressure, respiratory rate, blood oxygen saturation and body temperature. These five signals are generally considered essential to evaluate human health and a continuous monitoring should be made, mainly in patients. Ahrens in [12] presented two more vital signs that he considers important to be immediately measured if the patient is in any danger: capnography and stroke volume. Elliott and Coventry in [13] described another three important vital signs that should also be considered as part of routine patient assessment, such as pain, level of consciousness and urine output. According to these authors, the combination of these five additional signals with the five essential ones allows an accurate recognition of changes in patient’s physiology. Electrocardiography has also a major importance in heart electrical analysis, a very important tool to predict and diagnose cardiovascular diseases [14]. Glucose level monitoring is very relevant in humans with diabetes mellitus, a disorder where presently a huge effort in research is being made to develop non-invasive monitoring methods [15,16].

Beside these medical parameters there are also other important parameters to be monitored in order to evaluate neurological function, rehabilitation procedures, posture, motion control and sports performance, such as skin perspiration and actigraphy [15,16]. The next section discusses each of these signs, in terms of signal origin, medical and health importance and wearable sensors technology state-of-the-art. 

## 3. Valuable Vital Signs 

### 3.1. Electrocardiogram (ECG)

Electrocardiograms (ECGs) are among the most widely used biosignals, as a diagnostic tool in healthcare environment, providing information of the cardiac electrical cycle. The ECG waveform is characterized by five peaks and valleys (named P, Q, R, S, T, U), where each one represents a change in the electrical potential of the heart resulting in muscle activity and consequent in heart movement. The most differentiated peak of the ECG is the R-peak included in the QRS complex that represents the ventricles depolarization where there is a higher differential potential. Due to this reason, the consequent R-peaks (R-R interval) are used to measure the heart cycles [17].

The ECG waveform is used to analyse the cardiac rhythm, ischemic changes and to predict and treat acute myocardial infractions and coronary events. The analysis of the ECG waveform patterns plays a major role in the diagnose of cardiovascular diseases (CVD), such as atrial fibrillation, angina, atherosclerosis, cardiac dysrhythmia, congestive heart failures (CHF), coronary artery disease, heart attack, bradycardia and tachycardia [14,17]. One of the advantages of WHDs for medical purposes and with certifications in the ECG monitoring is the improvement of atrial fibrillation early detection due to longer continuous monitoring periods compared with a yearly single 24-h Holter.

Nowadays Ag/AgCl electrodes (wet electrodes) are the most commonly used to transduce ionic current from the heart into electron current in metallic wires. Its design and characteristics have in consideration cells low electrical potential difference (from −40 to −80 mV) and skin impedance. These electrodes are very reliable, compact and low cost, but can cause skin irritation due to its wet compound and adhesive properties. During longer periods of acquisition, the gel dries, resulting in a reduction of the contact between the electrode and the skin. Holters are the devices normally used for long acquisitions and their main disadvantage is the fact that it interrupts daily life routine, being unfeasible for unobtrusive continuous monitoring. To overcome this problem, fabric embedded electronics and dry electrodes have been developed using different materials [5,14]. This type of electrodes does not cause skin irritation, but, as they are not adhesive, artefacts appear due to body movement, making them clinically unfeasible for now. 

To reduce the artefacts produced by body movement and skin irritation, Luo et al. [18] developed a new technology of dry, flexible and stretchable sensors but still attached to the human skin, making it less viable to use outside the medical diagnose environment.

A possibility to overcome the adhesive properties is to have a dry electrode without adhesive properties resulting in proper electrodes for wearable applications. Chi et al. [19] developed a wearable, unobtrusive and patient friendly sensor that is able to acquire ECG signals through insulation such as fabric, being this technology firstly reported in 1969. Wearable electrodes are being embedded in textile fabrics where the fiber must be electrically conductive with low current and low impedance. These types of electrodes are normally a bundle of a normal fiber filament, like nylon, electrochemically plated with a metal or entwined carbon fibers. The fiber used in its fabrication must undergo several actions as stretching, bending and twisting, in order to adapt to the body movement, and to be washable [14,20].

Another type of ECG sensors are non-contact capacitive electrodes. These sensors are able to acquire the ECG data without direct skin contact but are more sensitive to motion artefact compared with conventional electrodes as reported by Aleksandrowicz and Leonhardt in [21].

### 3.2. Heart Rate (HR)

Heart rate (HR) is a standard vital sign and has become a routine measurement in both healthcare and fitness/sport activities. The monitoring of this signal provides information about the physiologic status by indicating changes in the heart cycle. This vital sign can be easily extracted from the ECG (R-peak) or photoplethysmography (PPG) signals [15]. Although these two physiological signs have different morphologic information in their waveforms and are from two different physiological origins, they contain a similar heart rate information. 

There are other ways to measure heart rate, like using inertial sensors [22] or scales [19], named ballistocardiogram (BCG), but are methods that do not have feasible measurement when compared with the HR extracted from the ECG and PPG.

It is very important in sport and activity contexts to evaluate or inform how the heart reacts during exercise and recovery. Heart rate variability analysis is gaining attention as a simple indicator of the health status of the cardiovascular system. It is also a human psychophysiological status indicator, such as in stress and fatigue measurements [12,23]. 

Medical professionals propose pulse signal as a measurement related to heart rate that can substitute it. It is defined as the palpable rhythmic expansion of an artery produced by the increase of volume of blood pushed into the vessel caused by the contraction and relaxation of the heart. This measurement provides more information than HR, like strength, amplitude and regularity of pulse. A problem referred in the literature is the decrease of blood volume in case of an irregular pulse or hypovolemia. Pulse signal should not be considered the same as heart rate and it can be estimated using pulse oximetry principles, method also used to measure blood oxygen saturation [14].

### 3.3. Blood Pressure (BP)

Blood pressure (BP) is considered the most important cardiopulmonary parameter, indicating the pressure exerted by blood against the arterial wall. BP provides indirect information about the blood flow when the heart is contracting (systole) and relaxing (diastole) and can also indicate cellular oxygen delivery. It is influenced by several human physiological characteristics: cardiac output; peripheral vascular resistance; blood volume and viscosity; and vessel wall elasticity. Ambulatory BP monitoring allows getting BP readings several times a day, which is ideal to monitoring high blood pressure (hypertension), one of the greatest threats to the global burden of diseases, improving cardiovascular diseases prediction [13,24].

BP is traditionally measured using inflatable pressure cuffs with a stethoscope on the patient’s arm. This method was adapted to perform autonomous BP measurement, including a fully automated inflatable cuff that measure BP by relating external pressure with the magnitude of arterial volume pulsations [5].

Continuous monitoring with a cuff can result in unwanted side effects, such as sleep disruption, skin irritations and an increase in stress levels. To solve this problem, new technologies for ambulatory BP monitoring have been developed [5]. One is to estimate BP based on pulse wave transit time between the pulse wave obtained by photoplethysmography (PPG) and ECG (R-peak), both measured on the chest [25] or with the PPG signal acquired on the wrist [16]. Yu-Pin Hsu and Young [26] developed a BP measurement new technique based also in pulse wave velocity but using two microeletromechanical sensors placed in two adjacent points of the body (wrist and neck). More recently Woo et al. [27] proposed an experimental watch-type prototype which uses a pressure sensor near the radial artery, giving an accurate blood pressure measurement on a personal smart phone, a real-time continuous monitoring BP wearable device.

### 3.4. Respiration Rate (RR)

Respiration rate (RR) is a fundamental physiologic parameter in patient’s observation. It is an accurate and important health information in several cases as it is in acidosis [13]. In critical illnesses, this is one of the most sensitive indicators such as in case of distress and potential hypoxia. Elliot and Convetry [13] state that the “respiratory rate is often not recorded in clinical settings or is simply guessed”, a major problem since RR is the best predictor of adverse events like cardiac arrest. In the medical environment, although there are multiparameter monitors, according to the authors the respiratory rate if often not recorded for analysis, such as ECG for example, and when these devices are not present, the RR is assumed (observation of the patient respiration during 30 s) or not accessed. According to Elliot and Convetry [13] a possible reason of this behaviour by medical specialists can be due the assumption that oxygen saturation provides a better reflection of patient respiratory function. Respiratory rate ambulatory monitoring is important in the detection of symptoms of respiratory diseases such as sleep apnea syndrome, chronic obstructive pulmonary disease and asthma, improving the administration of treatments if needed. This constant monitoring is particularly important in children with pulmonary diseases [13,15].

This vital parameter is normally calculated from the acquired respiratory waveform that reflects the chest volume variation during the inspiration and expiration. Thoracic expansion joined with muscle signs allow to calculate the respirator effort, indicating different physiological states. The analysis of these data in sport, mainly in competitive athletes can help in the achievement of a better respiratory performance [13,15,23].

Nowadays to obtain the respiratory function there are three primary methods: elastomeric plethysmography (EP), impedance plethysmography (IP) and respiratory inductive plethysmography (RIP). EP technique converts current variation of piezo-electric sensors in voltage using an elastic belt. Guo et al. [28] developed a prototype garment capable to measure chest and abdominal volume changes with high accuracy, using a piezoresistive fabric sensor. IP uses impedance changes of the body surface due to the expansion and contraction during breathing. This technology was used in the development of a uniform vest to be used in soldiers [4]. RIP technology principle is based on a loop wire with current that generates a magnetic field normal to the loop orientation. Chest volume variations change the area enclosed by the loop, creating an opposing proportional current [29]. Beside these three primary methods, other technologies are being used to get respiratory waveform: accelerometers [30]; extracted from the ECG signal [31]; derived from pulse oximetry [32] polymer-based transducers sensors [33]; optical fibers [34]; etc. Al-Khalidi in 2011 [35] has made a deep review about the methods used to measure respiration rate. Many other methods that are not suitable to be implemented in WHDs are referred on his review such as using infrared cameras or acoustic methods.

More recently, RR was acquired using a polymer named Polypower. This dielectric active polymer (DEAP) is being commercially produced as Polypower and changes its electrical attenuation when stretched in one direction. Before this type of polymers, the stretchable strain sensors were mainly based on fluid metals, such as mercury or gallium–indium, which was a dangerous material in case of a metal leak due to a possible packaging damage. The polymer based stretchable strain sensors allows to acquire electrical changes without the use of these dangerous fluid metals. Tognarelli et al. [36] are using this technology to obtain chest volume variations, showing the potentiality of DEAP in this area [36,37].

### 3.5. Blood Oxygen Saturation (SpO2)

Blood oxygen saturation (SpO2) is an extremely valuable vital parameter and easy to measure using photoplethysmography (PPG) technology and pulse oximetry principles. The PPG method enables to acquire blood vessel variation waveform, and when measured using two wavelengths (normally 660 nm and 905 nm) it is possible to estimate blood oxygen saturation. This is due to the haemoglobin absorbance spectrum change when it bounds with oxygen. Using oximetry principles it is possible to estimate the amount of oxygen that is being carried by blood cells (normally: 95–100%). This measure may lead to detect patient condition change that otherwise could be missed, such as lower percentage of oxygen (<95%) which indicates hypoxia and causes insufficient oxygen supply to the human body. One of the problems in blood oxygen saturation measurement is when the patient is anemic [13,38,39].

Besides medical use, pulse oximetry ambulatory monitoring has a particular interest in the evaluation of aerobic efficiency of a person undertaking a routine exercise. A study about the oxygenation of capillary bed from muscles can lead to a maximization of the athlete performance. Limb and brain oxygenation information is also important in military and space applications where gravity changes may affect the delivery of oxygen to these parts of the body leading to blackouts. It has been discovered the existence of a positive correlation between an individual’s performance and oxygenation responses as a function of task load [23].

There are several non-invasive technologies to measure blood oxygen saturation that can be applied to wearable devices, but PPG stands out being very popular in medical environment [38]. Finger is the most used place to acquire blood oxygen saturation levels and is the most commonly used in clinic conditions. Ring PPG sensors are under development due to its more wearing-comfortability and easily adaptation. Mobile connections leads these sensors to a much more independent and wearable device [40]. Ear lobe can also be used and a recent research presented a very small chip (3 × 6 mm) capable of measure blood oxygen saturation. PPG sensors in forehead are used to measure brain oxygenation and have already been developed as described in the literature [41]. A surface chest PPG reflectance prototype device was developed by Puke et al. [41] demonstrating the viability of oxygen continuous monitoring in this part of the human body. Recent developments made by Chen et al. [42] allows adjust PPG sensor parameters to refine the depth of tissue measurement, improving its functionality for clinical applications.

Concerning textile technology, several approaches have been developed to try to implement PPG sensors in this area. One approach is the integration of flexible plastic strips in weft direction containing two LEDs strips and two photodiode strips, with copper wires in the textile to conduct the signal in the textile fiber [43]. Another approach is to use optical fibers embroidered into textile. Krehel et al. [44] developed this new textile technology capable to analyze different depths of tissue, where both light-source and detectors are made using optical fibers.

### 3.6. Blood Glucose (BG)

Blood glucose (BG) is a worldwide measurement need in diabetic’s subjects. It is not measured in a normal procedure of a clinical environment, but is important in diabetic global population. According to the World Health Organization, in 2014 it was estimated that 9% of the worldwide population, aged above 18, had diabetes and in 2012 it was estimated 1.5 million deaths directly caused by this disorder. Diabetes disease causes several physiological disorders (cerebral vascular disturbance, retinopathy and nephropathy). To prevent it, diabetic individuals control blood glucose concentration measuring BG levels and inject insulin when needed to maintain the standard values. The most used method to evaluate BG concentration is collecting a blood sample by pricking the finger with a lancet. There has been a lot of effort to prevent finger pricking and as a result several devices have been developed and are already in the market that have the purpose to continuous measure BG levels still using invasive methods. Some examples are the Medtronic Continuous Glucose Monitoring (CGM) device capable to measure BG levels using an adhesive patch with a needle, sending the data wirelessly into a wearable insulin pump to release insulin into the human body [45]; Dexcom, Inc. (San Diego, CA, USA) has a device named Dexcom G4 Platinum also as an adhesive patch with a needle to measure the BG levels and is able to send the data wirelessly to a mobile device [46]. Dexcom become the first company to obtain FDA pre-market approval for their mobile application to support continuous monitoring. It can display data from their G4 Platinum CGM System, a system to measure BG from a needle with a sensor inserted just under the skin [47].

To try to prevent the human body invasion in BG measurements, non-invasive techniques have been developed to improve continuous self-monitoring and increasing efficacy in diabetes management during daily activities [16,48].

One of the firsts commercial wearable devices to continuous monitoring blood glucose was GlucoWatch^®^, providing glucose levels every 20 min during 12 h through the skin via reverse iontophoresis [49]. In 2007 it was discontinued due to skin burning effect. Other non-invasive techniques have been developed since then, such as: bioimpedance spectroscopy, a non-viable technique to continuous monitoring due to its poor reliability and acquisition requirements, where the user must rest 60 min before the measurements [50]; electromagnetic sensing with the disadvantage of being strongly affected by the temperature [50]; fluorescence technology [48]; mid- and near infrared spectroscopy are both possible technologies but presents several barriers, like weak penetration and reading correlation respectively; Measurement of BG through the eye, technology already used by Google to developed a prototype that has been taken to the FDA for early independent clinical trials [16]; Ultrasound has high sensitivity but with some interference from biological compounds [50]. These technologies have been already used in the development of several non-invasive BG wearable monitoring devices, like EyeSense and SCOUT. In some of these devices there are also temperature sensors, skin perspiration sensors and actigraphy sensors to predict energy expenditure, helping in the estimation of insulin that a subjects needs to administrate. There are still several challenges and barriers in this area, leading to a constant effort from many research groups to develop new technologies to get a stable, reliable, conveniently and economic continuous monitoring wearable device [49,50,51]. One of these difficulties is the delay of time between the concentration of blood glucose between the actual blood glucose and the one measured in the interstitial fluid, which may lead to a less reliable estimation of insulin dosage. To contour this issue new algorithms are being developed to estimate the future blood glucose levels with a time window of around 30 min [52].

### 3.7. Skin Perspiration

Skin perspiration is not a clinical parameter but a physiological sign used to analyze human reaction to several situations. Life situations can cause neurological reactions from the autonomic nervous system (ANS) stimulating an increase of skin sweating. This moisture changes the electrical conductance of the skin, allowing measuring the quantity of sweat produced by sweat glands, named as galvanic skin response (GSR). As ANS is responsible to control other physiological parameters like heart rate, respiration and blood pressure, GSR has been used alongside the acquisitions of some of these signals. For example, skin perspiration and heart rate variability can be used to classify mental states, helping in the distinction, as also in the detection of mental stress [53,54].

In sports, skin perspiration continuous monitoring is considered an important physiologic sign with enormous applications in this area and human behavior. It opens a new field of research in the clinical settings, such as in dehydration area, but it should not be interpret without knowing the physical activity context [15,54].

From skin perspiration, it is possible to obtain information about the physiological condition of the subject due to the several ions and molecules that constitutes it. For this reason, it is an excellent bio-fluid for non-invasive chemical sense to identify pathological disorders through ions levels, with a possible high benefit in clinical environment. Sodium, ammonium, calcium and lactate levels are indicators of electrolyte imbalance but also of cystic fibrosis, osteoporosis, bone mineral loss and physical stress. For example, physical stress can be used in psycho-physiological evaluation of militaries undergoing intense training [15,55].

There are two main types of sensors in skin sweat monitoring: epidermal-based sensors and Fabric/flexible plastic-based sensors:

(1) Epidermal-based have a conformal contact between the electrodes surface and the biofluid, like elastomeric stamps to print electrodes directly on human epidermis for continuous monitoring
(2) Fabric/flexible plastic-based sensors, the most used, having a main advantage of constant contact with a large surface area of the skin. These can be embedded into fabric or screen-printed into it, obtaining specifics measurements like pH and ions concentration as NH_4_^+^, K^+^ and Cl^−^ [55].

Kim et al. [53] have recently presented a new sensor to continuous measure GSR with high wearability and usability in a wide variety of fabrics. It is small, flexible and the surface is made of a dry polymer foam electrode to maintain a stable contact with the skin. A different technology is also under development—a technology based on microfluidic sweat analysis. Liu et al. [56] uses this technology, with a microcontroller and a Bluetooth module, to continuous monitor skin perspiration. Very recently, Koh et al. [57] have developed a epidermal sweat patch with microfluidic channels able to monitor sweat rate and uses biomarkers to monitor lactate, chloride, pH and glucose levels.

### 3.8. Capnography

Blood oxygen saturation measurement by pulse oximetry is a widely used method to access arterial oxygenation but it is not a good method for human ventilation assessment. Capnography is a non-invasive and cost effective method to evaluate human ventilation, indicating the carbon monoxide levels present in the respiration cycle, being very useful to avoid clinical problems and ensure patient safety [12,58].

Capnography continuously measures the inhaled and exhaled partial pressure of carbon dioxide (PCO_2_) in the respiratory gases, from where an estimation of the CO_2_ partial pressure in the arterial blood can be made. This measurement is made through air capturing just below the nose, where it goes to capnography device to perform CO_2_ gas quantification, obtaining a characteristic waveform from which it is also possible to obtain the respiration rate [58,59].

For more than 25 years, capnography has had a widely use in clinical practice as integral part of anaesthesia care in operating rooms, allowing anaesthesiologists to evaluate the consciousness level of the patient during the sedation process, but when patients are moved to the intensive care unit (ICU), they are not continuously monitored with capnography. A recent study shows a high correlation of morbidity and mortality with the underuse of capnography in ICUs [58]. Several reasons have been enumerated by Shankar [58] from Harvard Medical School to explain why capnography should be considered a routine monitor, highlighting that this method can be used as a guide to metabolic rate, or as an essential adjunct to monitor the integrity of airway, cardiac output and ventilation.

Capnography is mainly used outside the clinical environment to monitor sleep apnea syndrome. Normally this disorder is diagnosed and monitored using polysomnography or cardio-respiratory polygraphy (can also include capnography monitoring, but not as a single parameter), both high costs methods and dependent on the access to a specialized sleep laboratory. An investigation published in 2005 by Dziewas et al. [60] showed that capnography by its own can make an early diagnose of sleep apnea syndrome, helping to obtain a disease diagnose. A continuous monitoring is possible due to portable and wearable devices like MediByte from Braebon Medical Company that can be used at home with only 10% of a sleep laboratory usage cost [61].

Capnography is becoming a prevalent vital sign on portable devices and is helping first responders make life-saving decisions. In a near future, it is expected that capnography become widely used outside clinical environments. To ensure this, manufactures should make an effort to produce reliable, cost effective and portable capnography units with quick calibration procedures [58].

Another method to continuous asses PCO_2_ is transcutaneous capnography (TcCO_2_). Although it is less suitable to monitor patients during anaesthesia proceedings and it does not assess respiration rate, it has progressively shown good performance to monitor home ventilated subject as described in the study of Orlikowski et al. [62]. This method has been developed since late 1970s and it depends on the capillary blood flow increment by increasing the tissue temperature caused by a heating element in the electrode, assessing PCO_2_ using electrochemical electrode or oximetry principles. This method has revealed some issues related with calibration, elevated temperatures and associated burns. These sensor can take until 2 h to stabilize at regular human body temperature and to overtake this issue the sensor is heated to 42 °C or higher causing the mentioned associated burns [63,64].

### 3.9. Body Temperature

Body temperature (BT) is the outcome of the balance between heat production and heat loss in the body, being its measurement vital to avoid many elements defunctionalization due to high temperatures (e.g., proteins denature and lose function above certain temperatures) [23].

BT divides in two measures: core temperature (CT) and skin temperature. Skin temperature varies within a wider range of temperatures than core temperature, as the body’s thermoregulation mechanisms regulate core temperature. Skin temperature is affected by blood circulation and is also related with HR and metabolic rate [65,66,67]. External factors such as air circulation, ambient temperature and humidity also play an important role in this body temperature regulation mechanism [65,66].

Different wearable systems have been developed to measure both temperatures, such as skin-like arrays of precision temperature sensors or wearable adhesive devices to continuously measure temperature [68,69,70]. A very recent example is a re-usable wireless epidermal temperature sensor [71]—a battery-less RFID thermometer that is showing to be a promising device to estimate CT. 

Measuring CT through non-invasive methods, such as heart rate and skin temperature acquisition still remains a challenge. This is mainly due to the external factors that can affect physiological signs, making it difficult to have a direct correlation between these variables that only depends of the human physiologic thermoregulation mechanisms [66]. Looney et al. [72] presented a CT estimation algorithm based on hear rate and skin temperature (ECTemp^TM^) that according to the study of the authors, it can estimate at real-time the CT with a high level of accuracy that can be used as a thermal-work strain indicator in military populations. 

The gold standard for CT measurement nowadays is still the rectal temperature, and while other techniques like the telemetric pill allow for better usability, they face technical issues that influence the CT measurements. For instance, telemetric pill’s measurements are greatly influenced by the ingestion of hot or cold fluids [67,73].

### 3.10. Other Physiological Parameters

#### 3.10.1. Motion Evaluation

The evaluation of human body movements has several applications in medical rehabilitation, posture evaluation and sport performance. Motion analysis is widely used in actigraphy, a monitoring method to evaluate human rest and activity cycles that enables to provide an insight of daily activities routine. In medical rehabilitation, it is important to monitor mobility, in specific therapeutic exercises in order to evaluate movements and help in exercise techniques improvement, maximizing patients recover [16].

A WHD, with the proper sensors, can provide guidance and feedback to the patient and generate warnings based on the patient physiological conditions. Pulmonary rehabilitation can also be included in this type of monitoring, helping patient to complete a physical activity rehabilitation program. Posture is also an important factor that has a particular interest in patients submitted to hip surgery. Muscle activity can be acquired and associated to motor functional tasks. Motion evaluation and muscle activity used in sport activities allows accessing physiological signals, body kinematics and fatigue during exercise leading to athlete performance improvement. A recent example is reported by Maglott et al. in [74], where the arm movement timing is analyzed during basketball free throws [2,59,75].

To measure body movements, several sensors can be embedded in textile WHDs or in the portable units, such as inertial sensors (accelerometers, magnetometers and gyroscopes), electromyography electrodes, shoes force sensors [76] and even stimulators [2].

To obtain location and distance data, a GPS can be also added to the device. Complex body movement patterns can be measured, combining these sensors along with WHDs fabric, assessing to a higher amount of human body movements. The best way to access reliable data of human movements is using tight clothes. To contour this and try to obtain viable data using normal clothes, some research is being made to try to remove movement artifacts, enabling to use inertial sensors in casual clothes [77].

#### 3.10.2. Cardiac Implantable Devices

The increase of implantable cardiac devices is leading to a development of long-term surveillance, to improve patient safety and care. These devices are mainly implantable pacemakers, cardioverter defibrillators and cardiac resynchronization therapy systems. A remote monitoring of these devices will minimize the need of caregivers in several situations, allowing an early detection of adverse events and prompt corrective measure, accessing to up-to-date information stored in the devices memory. The incorporation of new communication technologies will provide a daily, remote, wireless, patient independent ambulatory monitoring of medical and technical data. A study with this type of system was conducted in a large population during four years concluding that, if the system was intelligently used, it was capable to improve care and increase safety of these patients [78].

### 3.11. Ambiance Parameters

Ambiance parameters are the environmental parameters in each subject surroundings and have a high relevance in several human body monitoring areas. The most used sensors are temperature, light, humidity and sound level. The continuous monitoring of air pollutants is also important due to its association with cardiac and pulmonary diseases.

Outdoor daily activities should also be continuously monitored with ambiance sensors to analyze ambiance characteristics that the human body is subjected during sport activities or simply rehabilitation exercises, being temperature and humidity important to evaluate dehydration [16].

With ambiance sensors it is possible to estimate the occupancy activity of subjects, easily estimating metabolic rate, mainly in indoors environments due to the non-contribution of external factors. According to a study made by Jin et al. [79] it is possible to distinguish several daily indoor activities through light, temperature and humidity measurement patterns.

Ambiance parameters such as ambient light, sound and temperature are also important to study and evaluate sleep quality and quantity. For example a sound sensor joined with an actigraphy sensor is an important tool to study sleep disturbances suffered by people living near airports [80].

## 4. Wearable Health Devices Generic System Architecture

Since the last decade, an aging population and the emergence of chronic diseases lead to a bigger interest in wearable physiological measurements devices. The effort in these developments is resulting in small wearable devices, with the benefits of a lower cost and higher mobility while the data is being collected [5].

**Figure 3:**
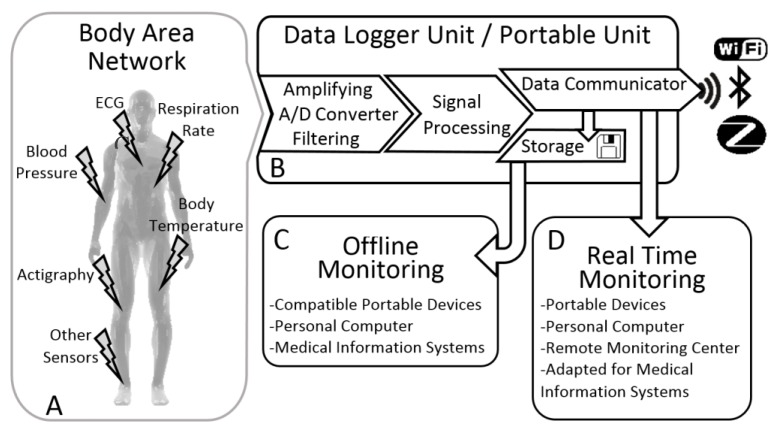
Generic architecture of wearable health devices system [3,10,81,82,83].

Based on our literature review we have designed an abstract generic WHD architecture (Figure 3) where we included the features from several wearable devices already developed both researched prototypes and commercial products. 

The generic architecture presented is divided in four modules: (A) Body Area Network, which can have different approaches, as we will see ahead; (B) Data Logger or Portable Unit whit all the electronic; (C) Data Analysis, an offline method to see the recorded data; (D) and Real Time Monitoring that enables to visualize live data [81,82].

### 4.1. Body Area Network

The term Body Area Network (BAN) can be sometimes confused with two other terminologies: Body Sensor Network (BSN) and Wireless Sensor Network (WSN). These three terminologies can be used in different types of wearable devices according to its architecture. In the image, the terminology BAN is used because it is related only with the sensors placed around the human body. The interconnection of these sensors creates a network of sensors (BAN), which are sent to a processing unit like a portable unit. If each node from the connecting network has a sensor or medical device with a sensing unit, containing more than just the sensor, we should refer it as a BSN rather than a BAN [10,12].

On the most general level, WSNs usually involve large numbers of low-cost, low-power and tiny sensor nodes, with each node having a predefined set of components (sensors, microcontroller, memory and radio transceiver [12], among others) granting that each node has sensing, computing, storage and communication capabilities [8].

Connecting all sensors by means of a network presents clear advantages, as it enables centralization of data in a single portable unit, gathering information from different sensors and sending it to external networks for remote processing. Furthermore, it enhances control, synchronization, scheduling and programming of the whole system, which allows the system to adapt according to present body condition and external environment. These advantages culminate in an optimization of the resources usage [10]. Wireless communication is a key asset and mandatory to enable systems to go mobile and ubiquitous.

### 4.2. Data Logger/Portable Unit

The portable unit (PU), also denominated as user interface box or datalogger unit, is where all the information is gathered, containing the outputs and inputs of the WHDs. The main inputs are the vital signs from sensors, but it can also receive data from other connected portable devices.

The communication between sensors and the PU is normally made through wires, resulting in a simpler and cheaper WHD. Some variations in this communication technique have been emerging, such as in smart clothes, where the interconnections (wires) are embedded and woven into the fabrics. This is a much more favorable approach in WHDs, avoiding loose wires around the body leading to a higher comfort and movement liberty. There is an innovative approach where the communication is made through biological channels, where the human body is used as a transmitter using electrostatic fields [5].

After receiving the analog vital signs, the PU amplify and/or filter the signals, converting then into digital signals. Signal processing can be made in the PU or in other device after the data transmission to it. This processing extracts features to evaluate the subject health, allowing the detection of an anomaly, the prediction of a disease or in the diagnose support. The raw-data collected by the PU can be transmitted using a wireless protocol or stored in a SD memory card. As Figure 3 shows, PU can also receive data from online monitoring devices and store it in a local memory. This two-way communication allow other devices to stablish a wirelessly connection to a main device, which stores the data of several sensors. This system can also be helpful to label the timing of important events using external devices [5,10,81,84].

The wireless protocols most popular in WHD are Bluetooth, Wi-Fi, ZigBee and more recently LoRa (Long Range radio). Bluetooth is a short-range radiofrequency based connectivity between portables and fixed devices requiring low-power consumption and with a low-cost. It is ideal for WHDs and widely implemented in commercial devices like smartphones and laptops. The new Bluetooth technology (version 4.0 and versions above) named Bluetooth Low-Energy (BLE) has even a lower power consumption with a smaller form factor, enabling an easier incorporation in small WHDs. Wi-Fi protocol lower layers were adopted, allowing higher data throughput for low-power requirements applications, not as low as the Bluetooth technology but can also be a good connection protocol to use in WHDs, mainly when a higher distance of communication is needed. ZigBee is another technology used for low power and low data rate communication protected by the use of the Advanced Encryption Standard. This feature makes ZigBee ideal to medical applications because it can consume less energy than Bluetooth versions earlier than 4.0, but with a lower data transferring rate. LoRa technology is a long distance coverage, low cost and low power consumption wireless protocol. According to Jeevan Kharel et al. [85] it is expected to overcome existing systems such as cellular technologies in the near future. It has the disadvantage of low data rate, but a huge advantage of scalability and customization of several parameters such as frequency channel, transmission power and data rate. This enables to reduce the power consumption and adapt it according to the transmission specifications [85]. Table 2 summarizes some of the main features of these wireless protocols.

Mobile telecommunications technologies can also be used to transmit real-time data using GPRS, a standard mobile data service in the global mobile communication. In the construction of a wearable device the communication protocol is very important in order to minimize the energy consumption [86]. A possible way to help battery longer lifetime is reducing the amount of data transmitted. With a data selection, it is possible to send it only when it is relevant, or save it in internal memory. At these cases a later offline data analysis can be performed. Data storing can be perform using SD memory card or internal digital memory slot, transmitting then data through a USB connection between the WHD and another device, where data from the SD card can be read. Another method to minimize the energy consumption is to incorporate compression techniques in the transmission protocol. Beside the reduction in the energy consumption this method help when network bandwidth limitations exists, or even when the storing space is reduced and a data compression is needed [87]. 

### 4.3. Real-Time Monitoring

A higher need of monitoring patients during long periods in the hospital led to real-time requirements. With WHDs it is possible to perform clinical monitoring outside a medical environment, alerting the patient in case of any physiological problem or just to monitor himself and be updated of his vital signs during daily activities [10].

In a medical environment WHDs allows the patients monitoring inside the boundaries of a specific area, normally a Hospital, where the patients can move while their vital information is being wirelessly transmitted to a remote monitoring center. Some devices can also send the patient location inside clinical environments. All these features allow the patient to move without any machines with him instead of being laid in the Hospital bed. These live systems can also be configured with a set of alarms for each patient helping in the detection of some required anomaly. This type of monitoring systems are defined by the literature as “Controlled Area” and “Wide Area” systems and always has a medical professional to provide information to the patients if needed. The vital signs can also be recorded in Medical Information Systems to be later analyzed by a medical professionals [81,88].

The biggest advantage of WHDs in real-time monitoring is the possibility of patient’s monitoring at home and outdoors, normally using regular internet communications at home and mobile network technologies in outdoors. This feature allows the patient to have a normal life while being monitored, with his vital signs continuously or intermittently transmitted to a remote monitoring center, with health support and, if needed, inform the patient of his medical status. The ambiance data acquisition is also important to know the conditions which people are exposed. For example, if an elderly person is subjected to an excessive cold or hot weather that can cause lung infection, dehydration or other diseases, an appropriate action might be taken to prevent a dangerous situation [15,81].

For last, vital signs can also be transmitted via Bluetooth to portable devices or personal computers to visualize and analyze the health status of an individual. This type of real-time monitoring can be used in sport activities to analyze the athlete vital signs during exercise, or in a simple daily run. This type of system is referred in the literature as “Wide Areas” and “Self-Monitoring” due to the non-involvement of medical professionals. Another example is the use of these devices in the health status monitoring of firefighters and combatants in field using mobile technologies, such as GPRS [81,84].

### 4.4. Offline Monitoring

All data from vital signs can be stored in a portable unit (micro-SD memory card for example), for future use in medical analysis or just as personal record. The data can be stored at the same time that a real-time monitoring is occurring. The main aim of such monitoring is to record vital data for clinic diagnosis and prediction by a medical professionals. For example, sleep issues such as apnea, can be analyzed through saved data from the patient: a home sleep monitoring allows to monitor sleep in a familiar environment resulting in reliable data acquisition [11,84].

## 5. Wearable Health Devices—Systems

Wearable health devices are becoming important ambulatory monitoring systems helping in medical prediction, anomaly detection and diagnosis. One of the problems is the limited number of measured physiological parameters. Many WHDs are developed with the aim to acquire only one physiological sign measurement, adapting its design and use to that only parameter. This fact results in several WHDs able to monitor a certain amount of vital parameters, but with monitoring limitations.

**Figure 4:**
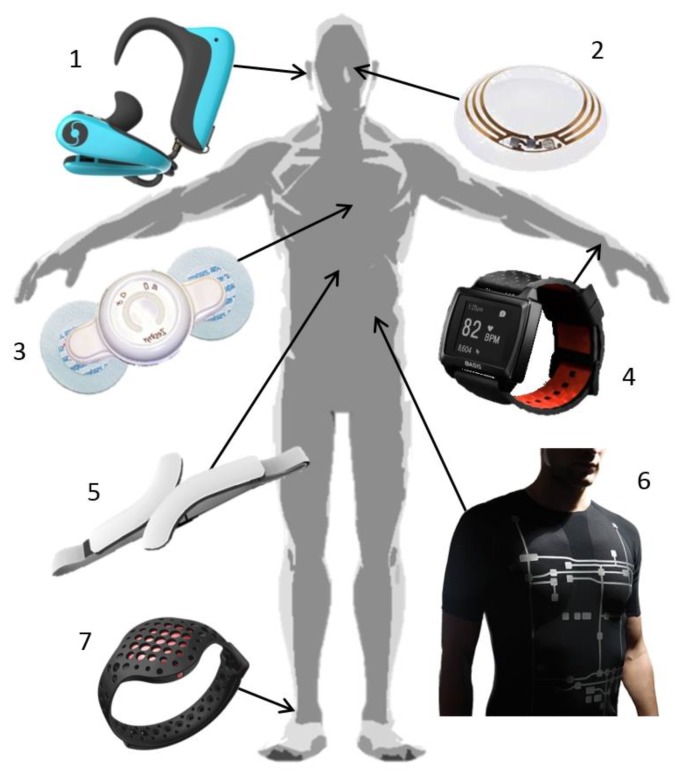
Examples of some wearable health devices. (1)—SensoTRACK ear sensor; (2)—Google Contact Lens; (3)—BioPatch^TM^; (4)—Smartwatch Basis PEAK^TM^; (5)—QardioCore; (6)—Vital Jacket^®^ t-shirt; (7)—Moov (activity tracker) [16,92,93,94,95,96].

**Figure 5:**
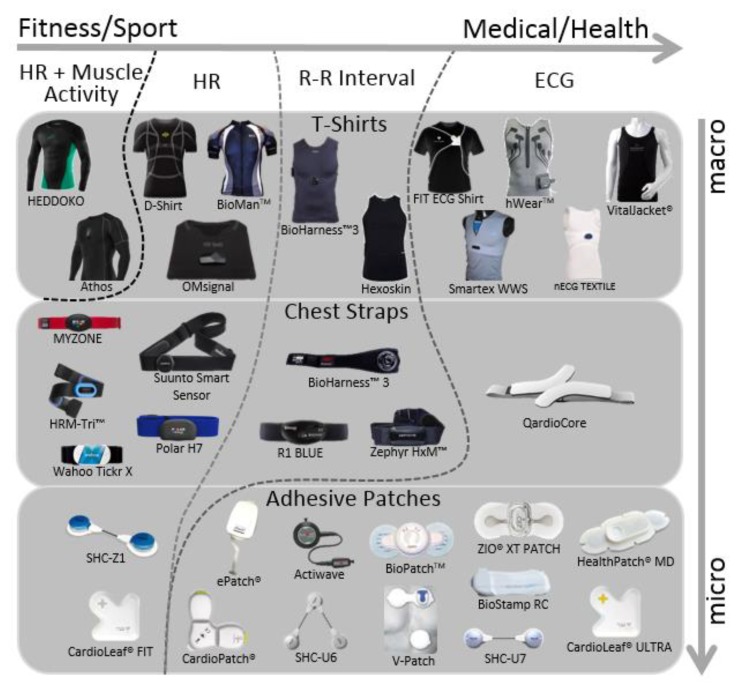
Heart activity trackers divided by type of WHD on the body (t-shirt, chest strap or adhesive patch) and purpose of usage (fitness/sport to medical/health). The closer to the medical/health side the higher the accuracy and quality of heart activity is. The characteristics chosen for this separation were based on published devices brand specifications [94,95,96,99,100,101,102,103,104,105,106,107,108,109,110,111,112,113,114,115,116,117,118,119,120,121,122,123,124,125].

**Figure 6:**
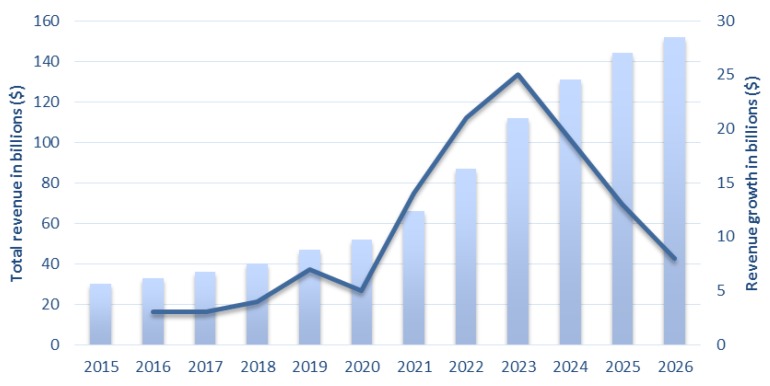
Horizontal bar graphic showing the total revenue in billions ($) (**left** axis) from 2015 to 2017, and estimated until 2026. The blue line shows the revenue growth rate in billions ($) (**right** axis). Adapted from [133].

**Figure 7:**
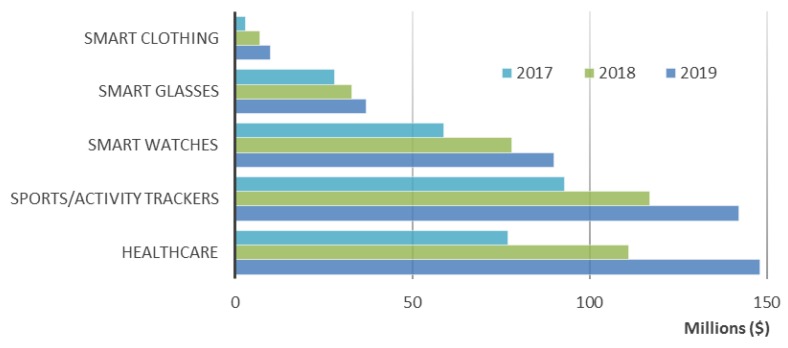
Horizontal bar graphic showing the trend of global market value of wearable computing devices, in millions, between 2017 and 2019. Adapted from [134].

Wristwatches, also known as smartwatches, are under development for a few years and are denominated as accessories for the human being. One of the first’s devices of this type was AMON, first presented in 2002 and capable of monitoring heart rate, blood oxygen saturation and skin temperature, already with a wireless data communication module [89]. More recently, a new smartwatches generation is emerging with wireless and mobile communication, able to provide more than 24 h of vital monitoring [90]. Their comfortable design, similar to a normal watch, allows to worn it constantly. These are being developed as activity and fitness trackers, monitoring physical activity like burned calories and distance travelled, heart rate, and, more recently, sleep monitoring, like PEAK^TM^ (Figure 4-(4)) which was the first smartwatch able to track sleeping cycles [91,92]. Moov (Figure 4-(7)) is a recent bracelet wearable to monitor movement. It can be used in different parts of the body according to the sport practiced, like swimming that is used in the wrist or in the leg in case of running activities [93]. 

Google eye lens (Figure 4-(2)) is a type of WHD that can represent the future of wearables, where they are going from macro size to micro and, in the future, will go nanoscale to be introduced in the body [16].

Another type of accessory device is emerging, an ear device that is able to acquire several physiological parameters like oxygen saturation level and heart rate. These type of devices, connected to the ear (Figure 4-(1)), are considered viable sensors due to its composition of mainly cartilage, removing muscle interfering, and has arteries near the surface. Valencell, a major supplier of sensing technology argues that ear signal is 100 times clearer than at the wrist. There are a very low number of these devices, and it can represent a new trend line of wearables [97].

A big group of wearable devices are related with heart activity. To continuously monitor this parameter there are three main types of wearable devices: chest straps (Figure 4-(5)); adhesive patches (Figure 4-(3)); and t-shirt (Figure 4-(6)) with embedded electronics. The first two are more capable to acquire several vital signs, but are not so comfortable and easy to wear as a simple part of clothing, like a t-shirt. When electronic technology meets garment the smart clothes denomination appears, being one of the best bets to acquire a higher amount of physiological signals since it covers a higher body area. To better analyse these three types of wearables (chest straps/adhesive patches/t-shirts) an analysis based on heart activity signal quality monitoring is going to be presented.

The incorporation of electronic based technology in garments lead to the concept of smart textiles. These can be applied to several areas from fashion (dresses with lights) to medicine (vital signs monitoring). A scientific effort is being made to evolve these smart fabrics into textiles capable to integrate unique properties that are normally performed by usual electronic systems, and can be divided in two categories: metal yarns incorporating conductive fibres or in electro-conductive yarns containing polymeric or carbon-coated threads. In terms of the development of smart textiles to be incorporated in WHDs, the main focus is on the textile electrodes (also called textrodes) to acquire the signals from the human body as it was already referred in some vital signs acquisition methods. It is possible nowadays to develop smart textile WHDs systems for a high number of applications for lifestyle and sport monitoring. This is an area where specific materials are needed, mainly in clinical application due to the requirements in terms of signal quality and device certification. For example, the heart activity monitoring is one of the vital signs where the smart textiles are playing a major role due to its importance. As already referred, the medical gold standard to acquire the cardiac activity waveform (ECG) are the wet electrodes, but due to skin irritation and discomfort the textile based electrodes can be an alternative solution compromising the signal quality and needing some preparations, such as skin cleaning and removal and hair. Nevertheless it is important to refer that although the skin sweat can lead to wet electrodes adhesive properties reduction, in the case of textrodes it is demonstrated that it increases local contact conductivity. There are different form factors textile-based WHDs to monitor the heart activity and a focus is going to be made to better understand these type of WHDs [20,98].

The quality on heart activity WHDs can go from a simple heart rate measurement to an ECG waveform quality signal. This depends of the signal quality that is being acquired, where the contact of skin with the sensor is a key factor. This in conjugation with the acquisition hardware device will determine the signal quality during subject movement, influencing the level of accuracy in the extracted signal [98]. According to our research, the devices can be divided in three types of heart activity accuracy: (1) HR- devices that acquires the R-peaks to estimate the heart rate, even if they are not capable to acquire all the consequent R-peak, they do an estimation of this value (unable to get ECG waveform); (2) R-R interval- devices that are unable to obtain the ECG waveform, but are able to determine the timing between each R-peak of the ECG signal; (3) ECG- devices capable to acquire ECG waveform, from which the morphologic parameters (ECG waveform peaks and valleys) can be extracted and analysed to perform cardiovascular diseases diagnose, cardiovascular rehabilitation analysis, among others (the heart rate is easily extracted from the waveform morphologic parameters—commonly the R-peak is used as a heartbeat marker).

Figure 5 presents a division of heart activity wearables monitor considering two approaches: type of wearable (chest straps, adhesive patches or t-shirts) and the purpose of the wearable usage, which goes from fitness/sp1ort to Medical/Health, where there is also an increase of heart activity accuracy (HR → R-R Interval → ECG).

From this analysis, we can see that an effort is being made in all type of heart activity trackers, with a higher relevance in adhesive patches. A problem in these multiple devices is their viability. This leads to questions of huge importance: which is the accuracy of each system? Does the respiration and activity movement influence the heart? R-R interval devices are viable when compared to devices that acquire ECG waveform and extract the R-R intervals from it? Is the ECG signal quality good? These questions could only be answered if all the devices were tested under the same condition. This would prove the quality of each system and the accuracy in heart beats detection. To try to investigate part of this subject a comparison study of the ECG t-shirts was made to evaluate the ECG quality and which certifications these type of devices offer nowadays.

### 5.1. ECG T-Shirt Devices

Based on the extensive search, we have identified five different t-shirt products capable of acquiring ECG waveforms: FIT Shirt from CardioLeaf^®^ [104], Smartex Wearable Wellness System (WWS) from Vivonoetics^®^ [105], hWear^TM^ from HealthWatch, nECG TEXTILE from Nuubo^®^ [106] and Vital Jacket^®^ from Biodevices, S.A. [94] (Figure 5). All of these devices have actigraphy trackers, internal storage and wireless communication. hWear^TM^, Vital Jacket^®^ and FIT ECG Shirt have at max 15, 5, and 3-lead ECG respectively, while the other two only have 1-lead ECG. An important thing to have in consideration is the type of electrodes: if they are wet or dry; and if they are textile based. This is an important fact that strongly affects the ECG waveform quality. A deeper analysis about electrode type VS quality in this type of devices is needed, including test all of them and compare the ones with wet and with dry electrodes in several activity indexes.

The best prove of WHDs quality is when these devices have medical approval. This fact proves that the system has medical quality. Medical approval increases system credibility, enabling it to be used in clinical environment or outside it, insuring the user that the data is as good as a stationary ECG signal recorder. With a quality ECG it is possible to get more information from the ECG waveform, better supporting the study of the human heart behaviour during daily activities. An example is the use of the ECG waveform changes in first responders to better understand their physiological state, such as stress and fatigue [126].

To enable a WHD to be used as a medical device, besides data quality in ambulatory monitoring it has to get clearance to be commercialized in the medical market. This depends from the country/region where the product is going to commercialized. Two examples are the European Union and the United States of America: in the European Union, these type of devices need a specific CE mark to be used as a medical device named the Medical Device Directive (MDD)—93/42/EEC. In the United States, Food and Drug Administration (FDA) is the agency responsible for medical product market clearance. Besides these certifications, there are also quality manufacturing regulations. The International Organization for Standardization (ISO) is the responsible for these regulations and the international quality of medical devices is under ISO13485 (ability to provide medical devices and related services) and ISO9001 (has a quality management system). These certifications ensure medical device quality, guarantying medical device quality during manufacturing processes. The following table (Table 3) resumes all the certifications and standards of each ECG t-shirt device.

### 5.2. WHDs Prototypes

WHDs development and design is not an easy task. Extract physiological data from the human body with the smaller WHD possible is a challenge, mainly when you try to combine several physiological signs and acquisition methods. Many WHD prototypes have been designed through the years with different technologies and approaches [127].

One of the first WHDs was the Georgia Tech Wearable Motherboard™, a project of the Technology’s School of Textile and Fiber Engineering financed by the US Department of Navy in 1996, resulted in a Smart t-shirt with novel fabric production process, able to measure temperature, heart rate and respiration. This WHDs was initially made with the purpose to be used in combat casualty care but then it could be adapted to different types of scenarios (e.g., first responders, astronauts, etc.), adding new characteristics such as ECG signal monitoring or hazardous gases. It had already a data real-time transmission via satellite.

The LOBIN device, presented in 2010, was a physiological monitor of the ECG, heart rate, angle of inclination, activity index and body temperature monitoring, tracking the location of the patients within the hospital. This device can store and transmit wirelessly vital data, supporting the configuration of alarms by setting different triggers for each patient. With LOBIN it was possible to monitor patient health status in real-time easily using a graphical user interface, which allowed the monitoring of several patients at the same time in the same screen, independently of the patient location [81].

The Sensing Shirt prototype was proposed in 2011 by Zhang et al. [128]. This prototype was capable to acquire basic cardiopulmonary parameters such as the ECG, abdominal and chest respiration, and blood oxygen saturation using PPG. All sensors, except PPG, are integrated in the shirt fabric and connected to a data acquisition unit by wires also integrated in the fabric. PPG sensor can be placed at the index finger/ear lobe and is plugged to the data acquisition unit through a cable and secure connector. This prototype has a 3-axis accelerometer to record body posture and activity, storing all the collected data in a micro SD memory card. In 2011 it was also presented a multi-parameter t-shirt capable to monitor the ECG signal, heart rate and respiratory rate by Sardini et al. [129], with a communication channel for remote assistance. This prototype has contactless ECG electrodes to avoid gels and skin irritations and all the data is transmitted by Bluetooth to a PC, being visualized or sent via internet for remote assistance. The authors defend the use of this device to home telemonitoring, evaluating vital parameters of the patient and his activity using a 3-axis accelerometer.

In 2013, a different sensorized shirt was presented by Farjadian et al. [130], named SQUID. This prototype was developed with the aim of home rehabilitation to monitor and evaluate physical therapy exercise with an online database for therapists’ remote supervision from a hospital. Although it does not monitor the ECG, it can monitor muscle activity using electromyography and heart rate, being capable to create an objective biofeedback so as to guide the user to follow the correct procedure as prescribed by the physical therapist.

Another t-shirt prototype was developed by Cafagna et al. [131] in 2014 and is called MyWear. This smart t-shirt has the disadvantage to measures only the heart rate, concerning heart monitoring, but it has an innovative respiration rate measuring technology based on PolyPower, a polymer that changes it conductivity when stretched as already referred. It also has a fall detection system and a plantar pressure distribution measurement system in a shoe. This prototype can connect to a smartphone for real-time monitoring functionalities and features visualization. Future research has the aim to validate the developed technology and platform.

## 6. Wearable Health Devices Market Trends

To perform a complete market analysis of WHDs, it is necessary to understand the market segments of these type of devices and then follow market lines to analyse specific markets. This approach will allow us to understand market values and trends in surrounding areas, which can also be possible target areas in a near future.

Wearable devices market value is in constant grow and this year it is estimated to reach a value of approximately $12 billion. It is a market that is in constant growth, if we think that in 2010 the market was only $6.3 million, it is possible to understand that in these recent years it has increased substantially (around 200 hundred percent) [132]. According to IDTechEx, in terms of global revenue, the following five year’s trend is to increase at a higher rate as it can be seen in Figure 6 [133].

Wearable devices market can be categorized according to the classification shown in Figure 7. According to this study made by ABI Research [134], from 2017 to 2019 the use of wearable devices in healthcare will constantly increase and can undergo the market value of wearables for sport/activity purposes. This fact is a good indicator for WHDs companies that have the aim to develop products for healthcare applications [134]. The smart clothing market that is still nowadays very small but its trend is to increase, reaching around $8.1 million in 2019 and is estimated to reach around $26 million in 2022 [135].

**Figure 8:**
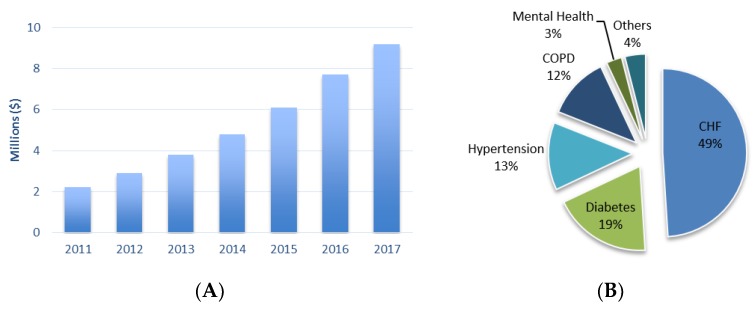
(**A**) Connected home medical monitoring devices (in millions) 2011 to 2017 [137]; (**B**) The world market for telehealth from 2014 divided in the main areas (CHF-congestive heart failures; COPD-chronic obstructive pulmonary disease) [138].

With the technology and internet of things (IoT) revolution, the healthcare wearable devices segment is increasing and with this the telehealth sector is also rapidly changing. In 2014 it was predicted that this year the revenues of telehealth devices and services reach $4.5 billion, which is almost the double of the 2017 value of $2.8 billion [136]. The number of home healthcare monitoring devices connected to a data center also has a growing trend considering the past years (Figure 8A) according to a study made by Berg Insight [137]. In this study it is possible to understand the evolution of this trend in the sector of home medical monitoring devices: diabetes care devices; blood pressure monitors; multi-parameter patient monitoring; apnea and sleep monitors; holter monitors; and heart rate meters. Although most of these device cannot have wearable features, it is possible to conclude that this is a continuous growing market segment, creating a market opportunity for the growth of WHDs in home healthcare for following years. 

Healthcare ambulatory monitoring segment, according to a study made by IHS Inc. (London, UK) [138] can be divided in several areas according to the diseases or the type of monitoring, resulting in five main areas: (Figure 8B): congestive heart failures (CHF); chronic obstructive pulmonary disease (COPD); diabetes; hypertension; and mental Health. This study conclusion is that mobile telehealth solutions are going to become the standard in remote patient monitoring, leading to a larger market for the WHDs. 

It is important to mention a fact regarding this market analysis: mobile health technology worldwide market in 2011 was about $1.2 billion and it is expected to reach an amazing $11.8 billion value this year, an important fact that supports the use of WHDs combined with mobile technology [139].

## 7. Conclusions and Future Challenges

Wearable health devices are a recent reality in healthcare and still under development with the aim to be integrate in medical health systems. There are some personal monitoring devices on the market capable to provide instantaneous single-parameter assessment and transmission. But the main value potential of WHDs is to integrate several biosensors, intelligent processing and alerts, to support medical applications, while interacting with health providers, using some of the technology that is not yet available on the market, only in research. The existing devices are highly expensive and work wireless based, technology that is not available everywhere in many countries, such as France where many consumers do not have access to broadband internet as reported in the literature in 2012 [15]. This type of data communication leads to another problem: privacy—a problem that concern healthcare, in the prevention of information, leading people to have lower confidence in these devices, resulting in ethical problems. This is one of the main barriers and the suggested solution is to create clear guidelines to providing privacy, confidentiality and proper use of electronic medical information [15]. A way to increase security of these data can pass by the use of personal non-clonable ID biometric technology to secure data, such as fingerprint, iris and ECG waveform [140]. All this effort will allow sharing secure data with a direct impact on societies, such as in China where WHDs may contribute to lower healthcare cost by introducing preventive healthcare strategies, cutting equipment and labour costs, eliminating unnecessary health services [15].

The next few years present a set of new interesting challenges in WHDs and to overtake them an adaption of these technologies should be made. It is important to study user requirements to develop an integrated approach in health and wellness services instead of devices and applications for single diseases. The development of such devices and its integration in an architecture of intelligent home services may have some main issues that require some attention: device efficiency, reliability and unobtrusiveness; privacy and ethical issues as already covered; legislation within and between states or countries; interoperability; end-user training to use WHDs; and social inclusion preventing users isolation, which can lead to a decrease of interaction with caregivers and physicians [11,15].

The good news is that the market seems to become more aware of WHDs possibilities and this will lead to more investment in R&D and better products to reach the market, hopefully enhancing the market growth in the coming years. 

In the sensor technology area, there are three main aspects that will have particular attention: long-term stability; resiliency; and biocompatibility. In textile-based sensors, washing cycles can cause mechanical, chemical and heat degradation, so an effort must be made to develop more resistant sensors to overcome stress caused by extended periods of use. Smaller sensors are also another important requirement, being associated to the electronics development, to increase functionality and portability of WHDs. The sensitivity of wearable sensors is an important characteristic that should be carefully analysed. A possible improvement may include the use of nanomaterial-based signal amplification. Biocompatibility must be highly considerate, covering sensors with antimicrobial or protective coating, preventing any potential toxicity of nanomaterials [55].

Wearable health devices are able to monitor a large amount of vital signs of human body, from advance sensor supervision in the case of infant respiratory to fitness applications or even soldiers on the battlefield. This particularity of WHDs creates a big excitement around this technology and many opportunities to continue its development. With new advances in new materials, electronics and telecommunication information technology, together with the entry of big multinational companies, such as Google, but also of small startups, WHDs are expected to overcome their challenges and enter in the consumer market with a higher impact in the following years [10,15,55].

## Figures and Tables

**Table 1:** WHDs Survey topics and restrictions.

Main Topic	Years Gaps	Purpose *	Vital Signs Words *
**Wearable Device**	2010–2013; 2014–2017	“Medical”, “Activity”	“Body Temperature”, “Blood Pressure”, “Respiration”, “Glucose”, “Heart Rate”, “oxygen saturation”, “Electrocardiogram”

* For each Purpose/Vital Sign a restriction in the search was included: all the other purpose/vital signs except the one that was being searched where added as a non-present topic to prevent papers duplication and to ensure that the searched papers were specifically about a topic.

**Table 2:** Wireless Protocols main features [9,85].

Wireless Protocol	Max Range	Max Data Rate	Power Consumption
**Bluetooth _(before version 4.0)_**	100 m	1–3 Mbps	2.5–100 mW
**Bluetooth Low-Energy (BLE)**	100 m	1 Mbps	10 mW
**Wi-Fi**	150–200 m	54 Mbps	1 W
**ZigBee**	100 m	250 kbps	35 mW
**LoRa**	50 km	700 bps	(customizable)

**Table 3:** MARKET CLEARANCE AND ISO STANDARDS OF EACH T-SHIRT DEVICE [94,104,105,106,107].

T-shirt Device	Market Clearance	ISO Standards
**nECG TEXTILE**	CE mark; MDD-93/42/EEC	ISO13485; ISO9001
**Vital Jacket^®^**	CE mark; MDD-93/42/EEC	ISO13485; ISO9001
**Smartex WWS**	-	-
**hWearTM**	FDA; CE mark	-
**FIT ECG Shirt**	-	-
